# Small Intestinal Bacterial Overgrowth Affects the Responsiveness to Colchicine in Familial Mediterranean Fever

**DOI:** 10.1155/2017/7461426

**Published:** 2017-12-12

**Authors:** E. Verrecchia, L. L. Sicignano, M. La Regina, G. Nucera, I. Patisso, L. Cerrito, M. Montalto, A. Gasbarrini, R. Manna

**Affiliations:** Periodic Fever Research Centre, Department of Internal Medicine, Policlinico Gemelli Foundation, Catholic University of Sacred Heart, Rome, Italy

## Abstract

**Objective:**

Familial Mediterranean fever (FMF) is an autosomal recessive disease due to a MEFV gene mutation. Since *Helicobacter pylori* infection has been described to increase the severity and frequency of FMF attacks, we evaluate if overgrowth of small intestinal bacterial (SIBO), associated with a release of bacterial products, can affect the response to colchicine in FMF patients poorly responsive to colchicine.

**Methods:**

We revised our Periodic Fever Centre database to detect FMF patients who were poorly responsive to colchicine, without a well-defined cause of drug resistance. They were evaluated for SIBO presence, then treated with decontamination therapy.

**Results:**

Among 223 FMF patients, 49 subjects show colchicine resistance, and no other known causes of colchicine unresponsiveness has been found in 25 patients. All 25 patients underwent glucose breath test; 20 (80%) of them were positive, thus affected by SIBO. After a successful decontamination treatment, 11 patients (55%) did not show FMF attacks during the following three months (*p* < 0.01), while 9 of them revealed a significant reduction of the number of attacks compared to three months before (*p* < 0.01).

**Conclusion:**

The SIBO eradication improves laboratory and clinical features of FMF patients. Thus, patients with unresponsiveness to colchicine treatment should be investigated for SIBO.

## 1. Introduction

Familial Mediterranean fever (FMF) is a rare disease due to mutation of the MEFV gene that encodes for *pyrin*, a protein involved in innate immune response regulation through interactions with the inflammasome, a macromolecular complex responsible for IL-*β*1 production and release. MEFV mutations result in an unbalanced control of flogistic response. Although considered a genetic hereditary disease, the diagnosis of familial Mediterranean fever is exclusively based on the Tel-Hashomer criteria, which are based on major criteria as the presence of high fever and serious pain, presence of amyloidosis, and effectiveness of colchicine and minor criteria as recurrent febrile attacks, erysipelas-like erythema, and a relative affected by FMF. Genetic tests can support but are not mandatory for diagnosis, for which is required the presence of 2 major criteria or 1 major and 2 minor criteria [1].

Colchicine is a fat-soluble alkaloid binding to *β*-tubulin, hindering its polarization with consequent inhibition of neutrophil chemotaxis and reducing expression of adhesion molecules. It prevents febrile attacks and is an FMF-controlling inflammation. Nevertheless, 5–10% of FMF patients are colchicine nonresponders. This condition may be due to concomitant diseases (vasculitis, inflammatory bowel disease (IBD)) [2, 3] or occult infections acting as trigger factors to reduce drug effectiveness [4, 5]. Chae et al. described that lack of pyrin induces hyperactivity of innate immune response against bacterial antigens such as lipopolysaccharides (LPS) [6]. MEFV-mutated pyrin is less effective than wild-type pyrin in binding to caspase 1 and therefore modulates immune tolerance against bacterial infections. In FMF patients, an increased reactivity to inflammatory conditions such as bacterial infections was observed. Indeed, some authors described cases of FMF patients with concomitant *Helicobacter pylori* (H.p.) infection showing more severe and frequent febrile attacks. Besides, a reduction of fever attacks and cytokine levels has been demonstrated after H.p. eradication [7, 8].

Small intestinal bacterial overgrowth (SIBO) is a condition characterized by the increase of microorganisms in the small bowel exceeding 10^5^ CFU/mL [9, 10]. It might be associated to peculiar anatomic and functional conditions leading to a defective host bacterial removal mechanism. SIBO may reveal through variable symptoms, from a complete malabsorption syndrome, with abdominal distension, dyspepsia, and diarrhea with or without pain, which is a colic type and modified by meals and evacuations of stools, to a total asymptomatic clinical presentation. It is important to emphasize that in spite of a possible overlapping of clinical frameworks, FMF and SIBO are different entities; FMF is indeed characterized by recurrent episodes of high fever associated with arthralgias and thoracic and abdominal pain, which is serous type, stabbing, and continuous. This pain is so important that it is easier to be confused with an appendicitis rather than colic pain.

Due to malabsorption and alteration of the intestinal flora, SIBO might facilitate blood diffusion of bacterial metabolic products, acting as PAMPS [11, 12] and interfere with many of the drugs' bioavailability [13].

Therefore, we hypothesized that SIBO may affect responsiveness to colchicine in FMF.

## 2. Aim of the Study

We assessed, through a longitudinal retrospective study, a SIBO prevalence in our colchicine-unresponsive FMF patients together with the effect of decontamination therapy on drug responsiveness.

## 3. Materials and Method

We evaluated our Periodic Fever Centre database between 1997 and 2014 to identify patients with FMF, diagnosed according to the Tel-Hashomer criteria [1, 14], who turned out not to be responsive to colchicine while taking an appropriate drug dosage (up to 0.03 mg/kg oral administration).

We excluded all patients with well-defined colchicine resistance [3], due to certain causes such as vasculitis and other autoinflammatory syndromes. Among the remaining patients, we enrolled only patients who have been evaluated for SIBO and then treated with decontamination therapy. As per conventional clinical practice, a month after the treatment, patients repeated glucose breath test to confirm the success of decontamination and the achievement of SIBO eradication. All patients were still taking colchicine at the same dosage used before decontamination.

In our center, all FMF patients starting a colchicine therapy usually undergo follow-up screening every three months for the first year of treatment; then, in case of responsiveness, it occurs every 6 or 12 months. Responsiveness to therapy is evaluated by physical and blood examinations, as reported below. In FMF patients who were responsive to colchicine, it was not considered necessary to screen for the presence of SIBO because they were asymptomatic.

### 3.1. Definition of Unresponsiveness to Colchicine

Unresponsiveness to colchicine is defined by FMF attack recrudescence, with usual features occurring more than once during three months since the beginning of the colchicine treatment, at maximum dosage of 0.03 mg/kg/day, according to the patient's characteristics [3].

### 3.2. Glucose Breath Test

To search the presence of SIBO, an H2 glucose breath test (GBT) was performed in the 20 patients left [15]. We verified that the GBT was performed under standard conditions:

- Patients should not have received antibiotics and/or laxatives in the month preceding the test.
- Subjects had a carbohydrate-restricted dinner on the day before the test and to be fast for the next 12 hours before the test, in order to minimize and to give stable values of basal H2 excretion.
- On the day of GBT, patients had rinse their mouths out with chlorhexidine 20 mL at 0.05%.
- Smoking and physical exercise were not allowed for 12 hours before and during the test; end alveolar breath samples were collected with a two-bag system immediately before and every 15 min for 2 hours, after having ingested 200 mL water isoosmotic solution with 50 grams of glucose [16].

According to the literature, GBT was considered indicative of SIBO presence when an increase of H2 levels over the baseline value was >12 p.p.m. with respect to the basal value [17].

### 3.3. Disease Activity Evaluation

Since the current literature shows no validate scales for disease activity under treatment, our PFC in daily medical practice refers to a questionnaire regarding some of the main features already used by Pras et al. [18] and Mor et al. [19], in their respective severity scores, in order to certify the responsiveness degree to colchicine three months after the beginning of the treatment. Particularly, we use Pras et al.'s score to evaluate the number of attacks in a one-month observation, and Mor et al.'s score for the presence of abdominal and/or thoracic pain, joint pain, attack severity, and limitations in daily life activities. Since some of the topics in our questionnaire make references to subjective parameters, a visual analogic scale (VAS) is used to determine the severity of abdominal, thoracic, and joint pain and daily activity limitation degree.

### 3.4. Blood Examinations

All patients followed up in PFC undergo blood examinations every three months, as objective parameters to evaluate the disease activity state. Particularly, acute phase reactants, like erythrocyte sedimentation rate (ESR), serum amyloid protein (SAA), and C reactive protein (CRP), are usually checked in outpatient regimen and then analyzed in Gemelli Polyclinic laboratories according to an internationally recognized standard methodology.

### 3.5. Decontamination Therapy

SIBO positive patients were treated with rifaximin 400 mg three times a day for seven days, according to scientific literature [20]. It was considered unuseful to treat SIBO negative patients to avoid unnecessary treatment.

### 3.6. Statistical Analysis

A paired sample *t*-test and NPAR tests and Wilcoxon signed-rank test were performed to analyze any changes in the clinical and laboratory features of FMF attack.

### 3.7. Ethical Aspects

No informed consent was necessary because anonymous retrospective data were collected during conventional clinical practices and analyzed according to the principles of the Helsinki Declaration.

## 4. Results

Among 223 FMF patients followed up in PFC, 49 subjects (M/F: 28/21; mean age 31.25 ± 9.35SD years; range 11–52) resulted poorly responsive to colchicine. Twenty-four of them were excluded for known colchicine resistance or for other concomitant diseases. In particular, 14 patients had H.P. gastric infection, 2 patients showed respiratory tract infection, 1 patient suffered from intestinal mycosis, 2 patients had IBD, 2 patients had vasculitis (Behçet's disease), 1 patient had TRAPS, 1 patient had marginal lymphoma, and 1 patient had bladder neoplasm.

Genotype of the selected 25 patients documented p.M694V homozygous for 2 patients, p.M680I homozygous for 1 patient, and p.V726A for 1 patient; 11 were heterozygotes (p.M680I, p.M694I, 2 p.V726A, and 2 p.M694V) among whom 5 complex heterozygotes (p.M680I-p.V726A, p.M694I-p.R761H, p.M694V-p.I692del, p.M694V-p.E148Q, and p.K695R-p.R202Q), 5 patients resulted in becoming carriers of polymorphism, and 5 had no MEFV mutations at all.

### 4.1. SIBO Prevalence in Colchicine Nonresponders

Among the 25 patients left, no other known causes for colchicine unresponsiveness were found. All patients underwent glucose breath test; 20 (80%) of them resulted positive, thus affected by SIBO, and 5 (20%) resulted negative.

### 4.2. Decontamination Treatment Response

SIBO positive patients underwent decontamination therapy with rifaximin at dosage of 400 mg three times a day. After one month, glucose breath test resulted negative in all those patients, meaning a complete decontamination.

### 4.3. Evaluation Disease Activity and Colchicine Responsiveness

#### 4.3.1. Clinical Features

**Figure 1:**
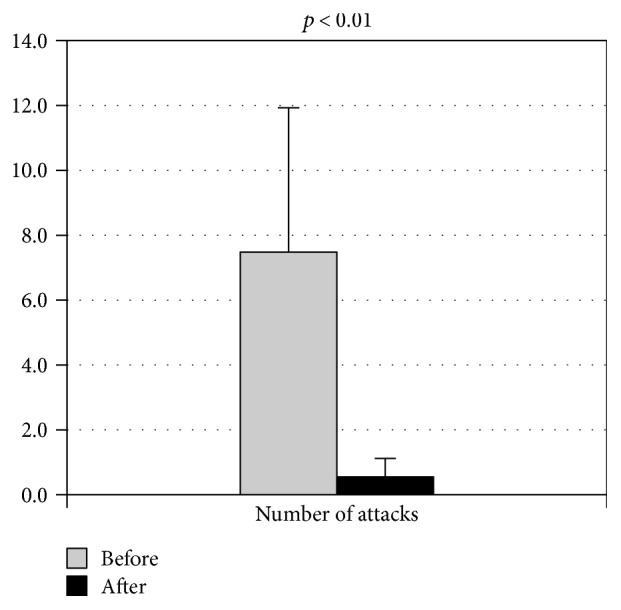
On the *y*-axis, the value of the number of attacks over three months before and after decontamination treatment.

Among the 20 patients resulting negative to glucose breath test control after the treatment, 11 patients (55%) did not show any FMF attacks during the following three months (*p* < 0.01), while 9 of them showed a significant reduction in the number of attacks when compared to three months before (*p* < 0.01) (Figure 1).

**Figure 2:**
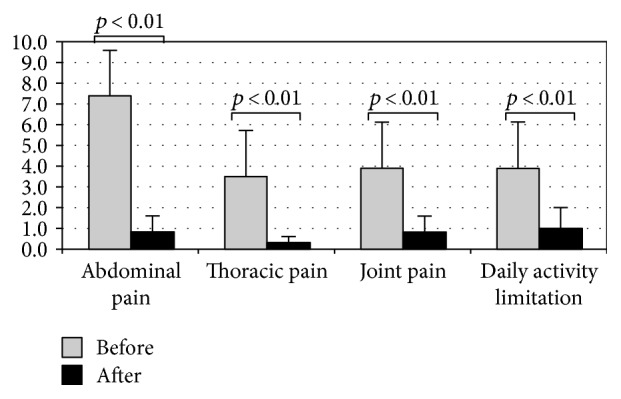
On the *y*-axis, the score obtained by VAS.

Each symptom analysis, evaluated by VAS, certified a significant decrease in abdominal, thoracic, and joint pains and daily life activity limitations after decontamination treatment (*p* < 0.01 for all voices) (Figure 2).

Severity disease reduction was reported in all patients showing a decrease of daily life activity limitation (Figure 2).

#### 4.3.2. Laboratory Features

**Figure 3:**
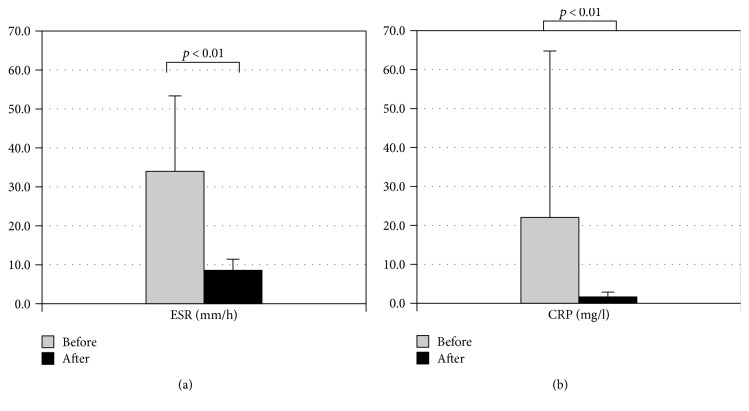
(a) Mean values of ESR before and after decontamination treatment. (b) Mean values of CRP before and after decontamination treatment.

Mean values of acute phase reactant were compared both before decontamination treatment (ESR 34.1 ± 20.8, CRP 21.8 ± 42.2) and after three months, showing a significant decrease (ESR 8.0 ± 3.2, CRP 2.1 ± 0.3) (*p* < 0.01) (Figures 3(a) and 3(b)). Reduction of inflammation parameters has been attributed only to the best control of basic autoinflammatory disease and not to the eradication of SIBO, since it does not induce elevations of systemic inflammatory markers but can cause elevation of local flogistic marker such as fecal calprotectin concentration [21].

## 5. Discussion

Considering FMF rarity but also the increased interest in natural immunity against bacterial products, we decided to verify retrospectively SIBO influence on colchicine responsiveness and on the clinical severity of the disease.

In our cases, we found a higher percentage of colchicine unresponsiveness than the one described in literature: 21.9% versus 5–10%. Nevertheless, there is still no consensus about its definition and no evidence is available regarding the management of this condition. The same definition of colchicine unresponsiveness shows some gaps, because it does not concern partial responsiveness. Indeed, there is no disease evaluation scale concerning treatment responsiveness. In order to find a proper solution, Ben-Chetrit et al. suggested to adopt the method of ACR 20, 50, and 70, so as to establish colchicine effectiveness evaluated on the reduction of FMF attacks per percentage each year, before and after drug administration. The author also made a clear distinction between “true” and “false” nonresponders based on the presence of some factors leading to such condition or the improvement of colchicine tolerance [22].

In fact, there might be various possible explanations at the basis of colchicine unresponsiveness. Lidar et al. in 2004 found a significant reduction of colchicine concentration in mononuclear cells (MNC) of nonresponders compared to responders. This difference was reported to less colchicine treatment compliance in the first group. For this reason, colchicine treatment failure in FMF patients was associated with an inadequate colchicine MNC concentration. Since this condition has been observed also in patients fully adhering to the treatment, it may probably result from a further genetic defect unrelated to the underlying FMF that may alter the drug concentration in MNC [23].

Inadequate therapeutic range of colchicine might also derive from an impaired drug absorption, as observed in SIBO in other diseases [24]. Indeed, the bacterial overgrowth could interfere with the normal adsorption of many substances such as carbohydrates, proteins, lipids, and vitamins; this condition may be due to bacterial fermentation of many sugars but also due to enterocyte injury. According to these considerations, the possibility of colchicine level dosage into MNC could be useful for a better understanding of drug unresponsiveness mechanisms. Unfortunately, in the clinical practice, the colchicine concentration assay is not currently available in MNCs in patients to know if they became responsive to therapy after decontamination treatment or because of better drug absorption.

On the other side, inadequate colchicine bioavailability in MNC might not be the only reason for drug unresponsiveness. Chae et al. [25] reported that *pyrin* gene mutations increase the flogistic response endotoxins, because MEFV mutated-pyrin is less effective than wild-type pyrin in binding to caspase 1 and inhibited it, leading to major activity of caspase 1 after Toll-like receptor activations and therefore to IL-1*β* production with the systemic inflammatory response to simple stimuli. Therefore, bacterial antigen production or release derived from SIBO may act as trigger factors, enhancing inflammatory cytokine production as IL-1*β* and sustaining a persistent or occult inflammation, producing an FMF phenotype apparently unresponsive to colchicine [11, 26]. We also considered the possibility of a possible anti-inflammatory activity of rifaximin, but we can exclude this hypothesis in the light of the scientific literature, which appears to be consistent in arguing that it is not absorbed systematically but exposes its function only at the local level.

In our series, acute-phase reactants were higher before decontamination therapy and decreased after rifaximin treatment. Indeed, all patients showed clear reduction of clinical, laboratory, and other disease activity parameters, restoring colchicine responsiveness. The result of this retrospective study based on the analysis of PFC registers, even if a control group is lacking, encourages to establish a multicentre prospective study in order to establish the SIBO prevalence in the general population and in the FMF population and also to confirm the role of SIBO eradication in FMF poorly response to colchicine.

## 6. Conclusion

We can conclude that SIBO affects the responsiveness to colchicine and the clinical severity in patients affected by FMF. We can assume that impaired intestinal bacterial products of intestinal microbiota may act in patients with innate immunity hypersensitivity as FMF or Crohn's disease, accentuating the clinical manifestations of autoinflammatory diseases. Second, we cannot exclude that SIBO may reduce the absorption of colchicine and cause a lack of its effectiveness.

On the basis of this study, we conclude that patients with FMF should be investigated for a suspected SIBO if presented with a reduced or absent responsiveness to the treatment with colchicine. In our study, bacterial decontamination restored the responsiveness to drug therapy and improved the clinical course of the disease.

Besides, this study suggests that intestinal microbiota modulate the clinical expression of the FMF and colchicine effectiveness. Moreover, these results have a major impact in terms of health economics, because improving the effectiveness of colchicine in patients with autoinflammatory diseases can reduce the use of more expensive drugs as biological agents.
